# Prevalence and risk factors of gammaherpesvirus infection in domestic cats in Central Europe

**DOI:** 10.1186/s12985-015-0381-6

**Published:** 2015-09-17

**Authors:** Reinhard Ertl, Melanie Korb, Ines Langbein-Detsch, Dieter Klein

**Affiliations:** VetCore Facility for Research, University of Veterinary Medicine Vienna, Vienna, Austria; Laboklin GmbH & Co KG, Bad Kissingen, Germany

## Abstract

**Background:**

Gammaherpesviruses (GHVs) are a large group of dsDNA viruses that can infect humans and several animal species. The two human GHVs, Epstein-Barr virus and Kaposi’s sarcoma-associated herpesvirus are known for their oncogenic properties in individuals with immunodeficiency. Recently, the first feline GHV, *Felis catus* gammaherpesvirus 1 (FcaGHV1) was discovered and frequently found in domestic cats in Australia, Singapore and the USA. FcaGHV1 is more likely to be detected in cats co-infected with the feline immunodeficiency virus (FIV).

**Findings:**

The prevalence of FcaGHV1 in pet cats from Germany and Austria was 16.2 % (95 % CI = 12.38-20.02). The odds for GHV infection were greater for FIV positive (OR = 4.5), male (OR = 13.32) and older (OR = 2.36) cats. Furthermore, FcaGHV1 viral loads were significantly higher in FIV-infected cats compared to matched controls.

**Conclusions:**

GHV infections are common in domestic cats in Central Europe. The worldwide distribution of FcaGHV1 can be assumed. A potential role as a co-factor in FIV-induced pathogeneses is supported.

**Electronic supplementary material:**

The online version of this article (doi:10.1186/s12985-015-0381-6) contains supplementary material, which is available to authorized users.

## Background

Gammaherpesviruses (GHVs) are a large and widely distributed group of double-stranded DNA viruses within the *Herpesviridae* family [1]. The two human GHVs, Epstein-Barr virus (EBV) and Kaposi’s sarcoma-associated herpesvirus (KSHV) are of particular importance due to their association with the development of cancer in immunosuppressed HIV patients [2]. In 2014, the first GHVs native to feline species (domestic cats, bobcats and pumas) were discovered in the USA [3]. *Felis catus* gammaherpesvirus 1 (FcaGHV1) was then frequently detected in domestic cats in Australia, Singapore and the USA [3, 4]. Interestingly, the prevalence of FcaGHV1 is higher in cats co-infected with the feline immunodeficiency virus (FIV) [4]. This feline retrovirus is a widespread pathogen in cats causing an AIDS-like immunodeficiency syndrome [5]. Furthermore, FIV-infected animals have a higher risk for the development of lymphoid malignancies, especially lymphomas [6]. However, the underlying mechanisms of FIV associated malignancies are not completely understood. Thus, similar to the human GHVs, FcaGHV1 in cats might be a co-factor for malignant transformation in immunocompromised individuals [7]. In this report, we assess the prevalence of FcaGHV1 among client-owned pet cats in Germany and Austria and evaluate potential risk factors such as FIV co-infection.

## Results

**Fig. 1:**
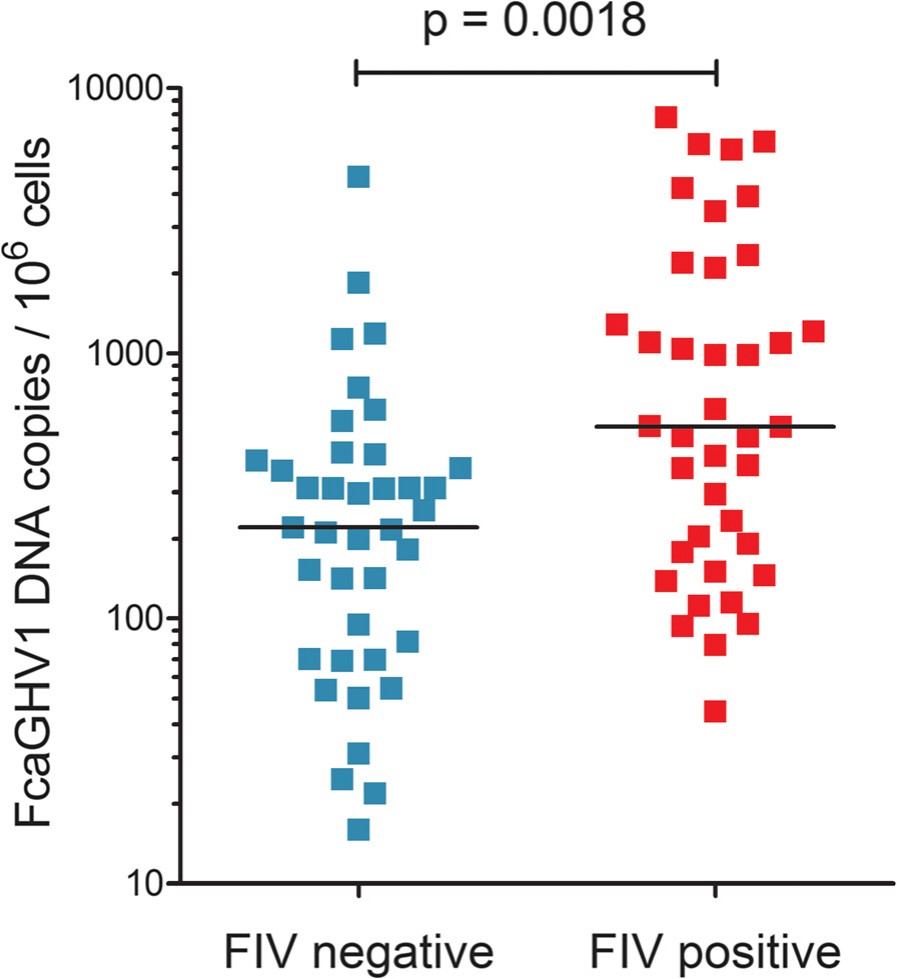
FcaGHV1 DNA viral loads are significantly higher in the blood of cats co-infected with FIV. Data obtained from FIV positive cats (n = 39), matched for age and sex to the FIV negative control group (n = 39)

A total of 462 cats from Germany (n = 402) and Austria (n = 60) were tested for the presence of FcaGHV1 DNA by quantitative PCR (qPCR) and FIV provirus DNA (qPCR and conventional PCR). The prevalence of FcaGHV1 was 16.2 % (95 % CI = 12.38-20.02) in FIV negative cats from both countries (n = 358). Among FIV-infected cats (all from Germany, n = 104) a co-infection with FcaGHV1 could be detected in 40.4 % (95 % CI = 30.95-49.81) of the animals. All tested Austrian cats were FIV negative and therefore excluded from the risk factor analysis. Odds ratios (OR) were calculated to evaluate risk factors for FcaGHV1 infection. FIV-infected cats in Germany showed a significantly higher probability for being FcaGHV1 positive (OR = 4.5) compared to non-infected animals (Table 1). Other factors that could be associated with FcaGHV1 infection were being male (OR = 13.32) and an increasing age (OR = 2.36). No association could be detected for the neuter status of the cats. Detailed clinical data were only available for the cats from Austria. These were grouped according to their health status (healthy vs. chronically ill). However, no significant differences could be found between the two groups. The numbers of FcaGHV1 and FIV DNA copies in the cat blood were measured by qPCR and analyzed for the individual groups. The FcaGHV1 viral loads were significantly (p = 0.0018) higher in FIV-infected cats (median FcaGHV1 viral load: 529 copies/10^6^ cells) compared to non-infected cats (221 copies/10^6^ cells; Fig. 1). Both groups have been matched for age and sex. FcaGHV1 copy numbers, however, did not correlate with the numbers of FIV copies in the co-infected animals (Spearman r = −0.024; Additional file 1). Parts of the FcaGHV1 glycoprotein B (gB) gene were sequenced from 12 German and 6 Austrian virus isolates. The gB sequences of all German and 5 Austrian samples (GenBank: KP862648, KP862649) were identical to those of previously reported strains from Australia and the USA [3, 4]. In one Austrian isolate (KT241042), a single, synonymous nucleotide polymorphism (adenine to guanine) was detected at nucleotide position 249 of the partial US gB sequence (KF840715) published by Troyer et al. [3].

**Table 1:** Prevalence and risk factors of FcaGHV1 infection in domestic cats in Germany and Austria; based on the results of chi square tests and the calculation of odds ratios

Factor	Category	Prevalence FcaGHV1	P value	Odds ratio	95 % CI	Origin
FIV status	Negative	39/298 (13.1 %)	Ref	-	-	Germany
	Positive	42/104 (40.4 %)	<0.0001*	4.499	2.684 – 7.541	Germany
Sex	Female	10/123 (8.1 %)	Ref	-	-	Germany
	Male	99/183 (54.1 %)	<0.0001*	13.320	6.553 – 27.07	Germany
Age	≤5 years	19/152 (12.5 %)	Ref	-	-	Germany
	>5.5 years	31/123 (25.2 %)	0.0066*	2.359	1.256 – 4.429	Germany
Neuter status	Male intact	17/63 (27 %)	Ref	-	-	Germany
	Male neutered	36/120 (30 %)	0.6691	1.160	0.588 – 2.289	Germany
	Female intact	3/55 (5.5 %)	Ref	-	-	Germany
	Female neutered	7/68 (10.3 %)	0.3288	1.989	0.489 – 8.087	Germany
Health status	Healthy^1^	8/31 (25.8 %)	Ref	-	-	Austria
	Chronically ill^2^	11/29 (37.9 %)	0.3130	1.757	0.585 – 5.279	Austria

CI = confidence interval, Ref = Reference category, * = statistically significant (p < 0.05), ^1^Includes cats examined for injury, intoxication or routine checkup, ^2^Cats diagnosed with chronic condition: cardiovascular disease (n = 6), tumor (n = 14), chronic kidney disease (n = 5), chronic inflammation (n = 3), neurological disorder (n = 1). Only animals with the relative information are enlisted

## Discussion

All samples analyzed in this study have been taken from pet cats during a veterinary medical examination. Clinical data were only available for a part of the animals. On account of this, only prevalence rates and already known risk factors for FcaGHV1 infection were evaluated [4]. The prevalence of FcaGHV1 among FIV negative cats in Germany and Austria (16.2 %) is similar to previously reported data from Australia (11.4 %), Singapore (9.6 %) and the USA (19.1 %) [4]. In comparison, FIV-infected animals from Germany were 4 times more likely to be tested FcaGHV1 positive. Furthermore, virus DNA loads were significantly higher in the FIV positive group. These results are congruent with previous reports [4]. Taken together, these findings indicate possible pathogenic interactions between FcaGHV1 and FIV, similar to the known associations of the human herpesviruses EBV and KSHV with HIV [2, 8]. In HIV patients, the virus-induced immunosuppression is presumed to generate an environment that facilitates the oncogenic properties of the GHVs. Thus, virally transformed cells cannot be properly eliminated by the immune system which eventually results in cell proliferation and the development of malignancies [8]. In this context, the increased FcaGHV1 viral loads in co-infected cats might be also a result of the FIV-induced immunosuppression, which possibly enables the reactivation of latent herpesviruses and subsequently enhances the replication of the co-pathogen. Other risk factors that were detected in the present study were related to the cats’ demographics. Male and older cats are more likely to be FcaGHV1 positive. These factors have been also described to increase the chance of FIV infection [9, 10]. No significant differences were detected between intact and neutered cats of the same sex. With regard to FIV, several studies reported a decreased risk of infection for neutered cats, while others found no significant effect of the neuter status [11–13]. When comparing healthy and diseased cats from Austria, we observed slightly greater numbers of virus-positive cats in the diseased group. Though, the statistical associations were not significant. In general, the determination whether a virus infection contributes to an animal’s disease status can be challenging in many cases. With the exception of lymphoid malignancies, clinical abnormalities found in FIV-infected cats are generally not significantly different to those of uninfected animals [10]. Additional studies with higher numbers of cats representing better defined disease categories are necessary to further investigate the pathogenic potential of FcaGHV1. Overall, striking similarities for the risk factors of the two viruses can be noted, indicating that both viruses share common transmission routes [4]. FcaGHV1 gB sequence comparisons of 12 German and 6 Austrian isolates with previously reported strains could detect one new nucleotide variation for this gene, found in a single isolate from Austria. As viruses with large dsDNA genomes are generally expected to have rather low mutation rates [14] and especially the FcaGHV1 gB gene is known to be highly conserved [3, 4], longer genome sequences will be needed to get deeper insights into the molecular relationship between different virus isolates.

In summary, our results show that GHV infections are common among European pet cats. Together with previous observations, this indicates that FcaGHV1 is most likely present in cat populations worldwide. FcaGHV1 detection was associated with male sex, increasing age and positive FIV status. Increased viral loads in co-infected animals support the assumption of pathogenic interactions between feline GHVs and FIV.

## Methods

EDTA blood samples were collected from pet cats presented to veterinarians in Germany and Austria in 2012–2015. All samples were taken as a part of the routine clinical examination. DNA was isolated from whole blood using commercial kits. FcaGHV1 qPCR and primers for DNA sequencing of the viral gB gene have been described [3, 4]. Bi-directional Sanger sequencing was performed by Microsynth, Balgach, Switzerland. The number of cell equivalents for each DNA sample was determined based on qPCR quantification of the feline RPP30 gene [15]. RPP30 copy numbers were used for the calculation of viral loads and as a control for the DNA preparation. The observed host gene numbers in the DNA samples ranged from 1.17 × 10^4^ - 1.72 × 10^5^ copies RPP30/2 μl, which was found to be sufficient to reproducibly detect also low levels of viral DNA. The detection limit of the virus-specific qPCR assay, which was evaluated by a dilution series of FcaGHV1 gB-containing plasmid DNA, was 2 copies per reaction. All cats were tested for FIV provirus DNA by qPCR and conventional PCR as previously described [16, 17]. Plasmid DNA standards were prepared for all qPCR assays (FcaGHV1, FIV1416p and RPP30) to quantitate the DNA copy numbers. Statistical analyses were performed using the GraphPad Prism 5 software (GraphPad Software, Inc.). A chi-square goodness of fit test and odds ratios were used to compare prevalence rates between the groups. FcaGHV1 viral loads were compared using a two-sided Mann Whitney *U* test. Spearman’s rank correlation coefficient was calculated to assess the relationship between FcaGHV1 and FIV viral loads. Statistical associations were considered significant at p < 0.05.

## Additional file

Additional file 1:
**No correlation of FcaGHV1 and FIV DNA copy numbers in the blood of co-infected cats.** Virus copy and cell numbers were measured by quantitative PCR. Spearman’s rank correlation coefficient was calculated to assess the statistical association. (TIFF 10392 kb)

## Abbreviations

CI: Confidence interval
EBV: Epstein-Barr virus
FcaGHV1: *Felis catus* gammaherpesvirus 1
FIV: Feline immunodeficiency virus
gB: glycoprotein B
GHV: Gammaherpesvirus
KSHV: Kaposi’s sarcoma-associated herpesvirus
OR: Odds ratio
qPCR: Quantitative PCR
