# Supplementary figures and images for: Prevalence and risk factors of gammaherpesvirus infection in domestic cats in Central Europe
Source: Virol J. 2015 Sep 17;12:146. doi: 10.1186/s12985-015-0381-6 (PMC4574186; doi:10.1186/s12985-015-0381-6)

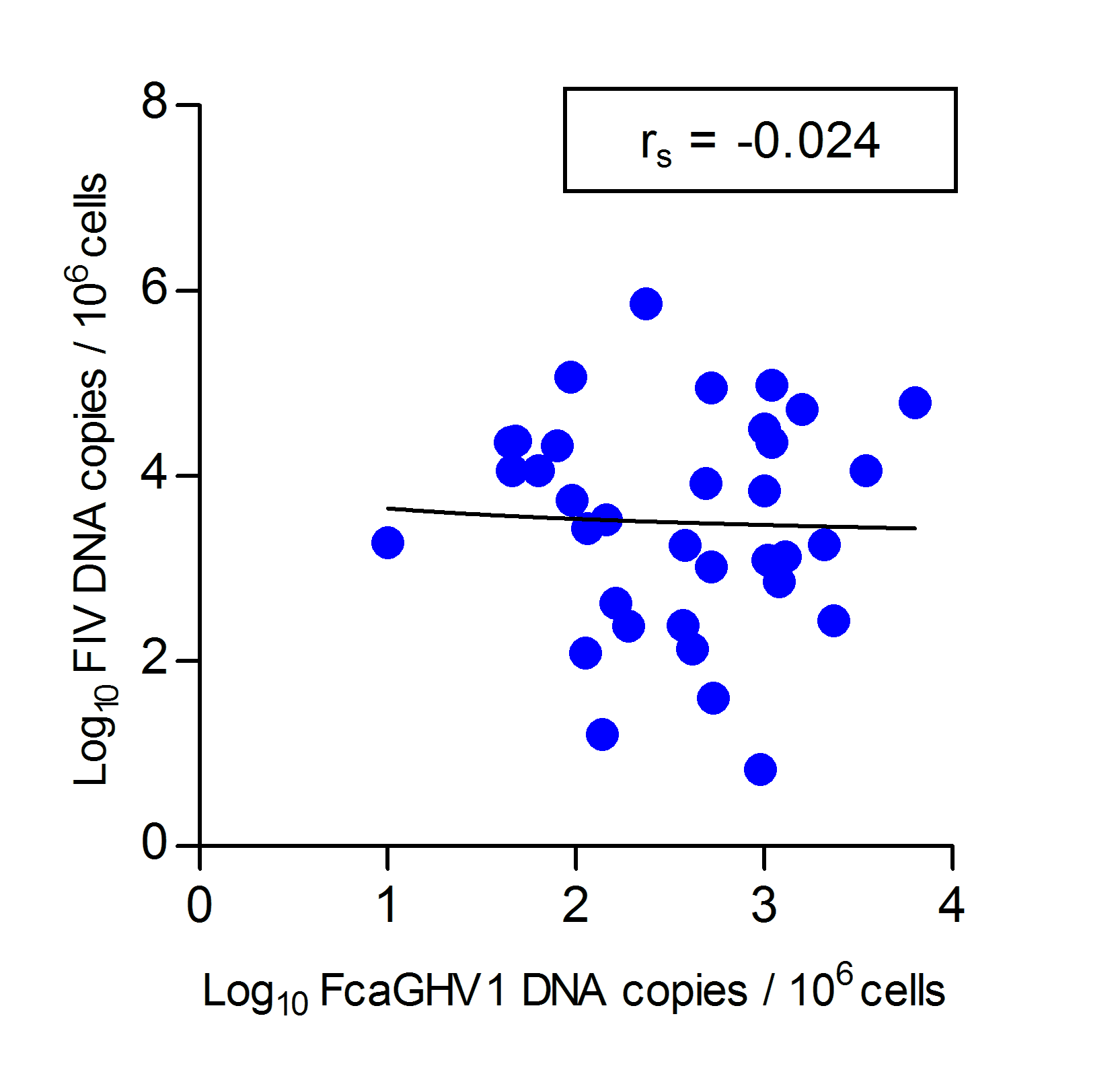

Supplement: Additional file 1: — No correlation of FcaGHV1 and FIV DNA copy numbers in the blood of co-infected cats. Virus copy and cell numbers were measured by quantitative PCR. Spearman’s rank correlation coefficient was calculated to assess the statistical association. (TIFF 10392 kb) [file 12985_2015_381_MOESM1_ESM.tif]
